# Understanding physical activity intentions among French Canadians with type 2 diabetes: an extension of Ajzen's theory of planned behaviour

**DOI:** 10.1186/1479-5868-6-35

**Published:** 2009-06-16

**Authors:** François Boudreau, Gaston Godin

**Affiliations:** 1 Department of Nursing, Université du Québec à Trois-Rivières, G9A 5H7, Canada; 2 Canada Research Chair on Behaviour and Health, Faculty of Nursing, Laval University, Pavillon Ferdinand-Vandry, 3e étage, 1050 rue de la Médecine, Québec (Québec), G1V 0A6, Canada

## Abstract

**Background:**

Regular physical activity is considered a cornerstone for managing type 2 diabetes. However, in Canada, most individuals with type 2 diabetes do not meet national physical activity recommendations. When designing a theory-based intervention, one should first determine the key determinants of physical activity for this population. Unfortunately, there is a lack of information on this aspect among adults with type 2 diabetes. The purpose of this cross-sectional study is to fill this gap using an extended version of Ajzen's Theory of Planned Behavior (TPB) as reference.

**Methods:**

A total of 501 individuals with type 2 diabetes residing in the Province of Quebec (Canada) completed the study. Questionnaires were sent and returned by mail.

**Results:**

Multiple hierarchical regression analyses indicated that TPB variables explained 60% of the variance in intention. The addition of other psychosocial variables in the model added 7% of the explained variance. The final model included perceived behavioral control (β = .38, p < .0001), moral norm (β = .29, p < .0001), and attitude (β = .14, p < .01).

**Conclusion:**

The findings suggest that interventions aimed at individuals with type 2 diabetes should ensure that people have the necessary resources to overcome potential obstacles to behavioral performance. Interventions should also favor the development of feelings of personal responsibility to exercise and promote the advantages of exercising for individuals with type 2 diabetes.

## Background

In Canada, considering the increasing incidence of type 2 diabetes [1], it is estimated that this disease and related complications [2] will soon represent an important challenge for health professionals and public health authorities. Fortunately, for almost all individuals with type 2 diabetes, improved glycemic control would be sufficient to prevent or delay micro- and macrovascular complications. This could be accomplished by the practice of regular and moderate physical activity [3]. According to Canadian guidelines, individuals with type 2 diabetes should accumulate at least 150 minutes of moderate-intensity aerobic exercise each week, spread over at least three non-consecutive days of the week [4]. It is estimated that Canadians with self-reported diabetes are more likely to be physically inactive (65%), compared to members of the general population (56%) [5]. Therefore, identifying strategies that facilitate participation in physical activity for individuals with type 2 diabetes represents a major challenge for health educators.

In the field of health promotion and prevention, it is increasingly recognized that the development of effective educational interventions requires an understanding of the phenomenon being studied for a specific population in a given context [6]. Moreover, the selection of theories is an important step in developing interventions [7]. In this regard, social cognitive models can prove helpful in understanding key determinants of a given behavior.

One of the most thoroughly tested social cognitive models is Ajzen's Theory of Planned Behavior (TPB) [8]. It has been applied to understand several health-related behaviors [9], including exercise or physical activity [10,11]. A meta-analysis [10] showed that the TPB variables accounted for, on average, 30.4% of the variance in intentions to exercise. According to the TPB, the proximal determinants of intention to adopt or not to adopt a behavior are attitude, subjective norm and perceived behavioral control with respect to adopting the behavior. Attitude represents an individual's evaluation of the perceived benefits and drawbacks of adopting a given behavior (e.g. "My doing physical activities in my free time during the next month would be good/bad"). Subjective norm reflects the perceived expectations of specific individuals or groups regarding the adoption of a given behavior (e.g. "In your opinion, are the people who are most important to you in favor or not in favor of your regular participation in one or more physical activities in your free time during the next month?"). Lastly, perceived behavioral control is determined by the individual's perception of the presence or absence of resources and opportunities, as well as perceived obstacles and impediments regarding adoption of the target behavior (e.g. "To me, participating in one or more physical activities in my free time during the next month appears difficult/easy"). In addition, each of the TPB variables is defined by a set of salient beliefs. These three sets of beliefs are *behavioral beliefs *(attitude toward the behavior), *normative beliefs *(subjective norm), and *control beliefs *(perceived behavioral control).

Recently, two studies tested the TPB [12] to understand physical activity among individuals with type 2 diabetes. White et al. [13] used a cross-sectional design to examine exercise beliefs among Australians with type 2 diabetes or cardiovascular disease (n = 192). Multiple regression analyses indicated that exercise beliefs explained 12% of the variance in behavior. However, White et al. did not report the proportion of variance explained in intention. For their part, Plotnikoff et al. [14] tested the TPB in order to provide guidance for the development of programs aimed at promoting physical activity among Canadians with type 1 (n = 697) or type 2 diabetes (n = 1614). In a cross-sectional model, the results indicated that 40% of the variance in intention for those with type 2 diabetes was explained by attitude (β = 0.36, p < 0.05), subjective norm (β = 0.12, p < 0.05), and perceived behavioral (β = 0.34, p < 0.05). Respectively, intention (β = 0.23, p < 0.05) and perceived behavioral control (β = 0.09, p < 0.05) explained 8% of the variance in physical activity (6 months). Unfortunately, Plotnikoff et al. did not identify the beliefs underlying the main variables of the TPB. Therefore, the information needed to develop an intervention is missing.

Past research in the determinants of physical activity suggests that determinants other than those identified in the TPB can contribute to explaining additional portions of variance in intention [15]. In this respect, four determinants must be considered: anticipated regret, moral norm, descriptive norm and past behavior. According to Abraham & Sheeran [16], anticipated regret "refers to beliefs about whether or not feelings of regret or upset will follow inaction (e.g. 'I would regret it if I did not exercise tomorrow')" (p.496). In the field of physical activity, Abraham & Sheeran [16,17] observed a correlation of .57 between anticipated regret and intention among a sample of undergraduate students. In a similar study, Conner & Abraham [18] reported a correlation of .48. Abraham and Sheeran [17] also observed that anticipated regret contributed to a substantial increment in the variance of intention (5.3%), after the determinants of the TPB and past behavior were taken into account. More importantly, however, was the demonstration that an intervention designed to highlight anticipated regret towards not exercising significantly enhanced exercise intentions [17]. Thus, it is justified to verify if anticipated regret contributes to the prediction of intention among individuals with type 2 diabetes.

Moral norm measures "feelings of personal obligation towards the adoption of the behavior" [19]. A growing body of research supports the inclusion of this determinant in the prediction of intention [20]. The additional explained variance in intention, after the TPB determinants are considered, is about 4% [15]. Similar findings are observed in the field of physical activity [21,22]. This determinant could play a significant role in strengthening intentions to exercise among individuals with type 2 diabetes, since most individuals with type 2 diabetes know about the importance of exercising for their health and of their responsibility in this regard [23]. It is thus possible that a recommendation to exercise by a health professional increases feelings of moral obligation to take responsibility for personal health.

Recently, it was recommended that a descriptive norm as a determinant of intention [24] be added. A descriptive norm "refer to an individual's belief about how widespread a particular behavior is among referent others" [25]. It is hypothesized that as the perceived prevalence of a given behavior increases, the likelihood of intent to adopt this behavior increases [25]. In a meta-analysis comprising twenty-one tests of the relationship between descriptive norm and intention, the addition of descriptive norm to the prediction of intention, after the TPB determinants had been taken into consideration, led to a significant increment of 5% in the explained variance [26]. With respect to physical activity, studies among samples of undergraduate students [27,28] and cancer survivors [28] observed that descriptive norm was a significant independent predictor of intention. Therefore, it is appropriate to verify the relevance of descriptive norm among individuals with type 2 diabetes.

Finally, Conner and Armitage [15] showed that, after taking into account the TPB determinants, past behavior explained, on average, a further 7.2% of the variance in intention. Past behavior was retained on the basis of a meta-analytic review, indicating that it may play an important role in predicting intention to be physically active[29].

### Purpose of This Study

The aim of the present study was twofold: to identify the determinants of intention to participate in leisure-time physical activity among French Canadians with type 2 diabetes and to identify the beliefs underlying the determinants of intention that could be used to develop an intervention. The following hypotheses were formulated based on Ajzen's Theory of Planned Behavior and earlier research: (1) Attitude, subjective norm and perceived behavioral control will constitute significant determinants of physical activity-related intentions; (2) Anticipated regret, moral norm, descriptive norm and past behavior will explain a significant amount of additional variance beyond the direct determinants of the TPB in the prediction of intention.

## Methods

### Participants and Procedure

A total of 2000 individuals with diabetes (age range 35–64 years) were randomly selected from the data sets of the « la Régie de l'assurance maladie du Québec » (Quebec Health Insurance Board). This data source does not differentiate between type 1 and type 2 diabetes. Self-reported questionnaire used in the present study allowed the distinction between these two types of diabetes. Authorization from the "Access to information Commission" was obtained and all study procedures were approved by the University Ethics Committee. A package was mailed to the individuals containing a self-administered questionnaire, return envelope, and cover letter – signed by the investigators – describing the purpose of the survey and requesting the individuals' cooperation. Recipients were advised that their responses would remain confidential and that no name would appear on the questionnaire. One follow-up reminder was sent 10 days after mailing the initial questionnaire. All questionnaires used were in French-language.

Of the 2000 questionnaires mailed, 144 were returned because recipients were not interested in participating in the study, 92 self-reported major physical limitations in terms of physical activity, eight had incorrect addresses, three did not understand French and two died before receiving the questionnaire. Overall, 588 completed questionnaires were received for a participation rate of 33%. Of these, 33 (5.6%) did not answer the question on self-reported diabetes, while 54 (9.7%) and 501 (90.3%) were individuals with self-reported type 1 and type 2 diabetes, respectively. For the purpose of our study, only individuals reporting type 2 diabetes were retained.

### Variables

An elicitation study was first conducted to identify modal salient beliefs. Participants (n = 37) were individuals with type 2 diabetes aged 35 to 64 years and participating in a four- to five-day health education program on diabetes self-management. Two methods were used to obtain this information: a semi-structured focus group (n = 6), where all verbatim statements made by participants were recorded and entered into an electronic database; and an open-ended questionnaire (n = 31). The mean age of these 31 participants was 58. 2 years (SD = 9.7) and 52% were male. Regardless of the method, participants were asked about: (1) advantages and disadvantages of «*participating regularly in one physical activity or more during their free time in the next month *»; (2) persons or groups of persons who think that they should or should not perform the behavior in question; and (3) the things that made it easy or difficult for them to practice one or more physical activities on a regular basis. Following each of these procedures, the first author of the current study and a research assistant independently identified the salient beliefs to be used in the questionnaire.

The psychosocial determinants assessed were the variables of the TPB as well as anticipated regret, moral norm and descriptive norm. As recommended by Ajzen & Fishbein [30], psychosocial determinants were defined in terms of *action *(participate regularly), *target *(in one or more physical activities), *context *(in my free time), and *time *(during the next six months). Table 1 describes each of the psychosocial variables. The questionnaire was submitted to a test-retest procedure over a two-week period to a subgroup of 30 individuals with type 2 diabetes. The mean age of participants was 58.9 years (SD = 9.8), 33% were male and 33% had completed postsecondary education. Test-retest intraclass correlation coefficients and the Cronbach's alpha coefficients were verified and are reported in Table 1.

**Table 1:** Description of the psychosocial variables and psychometric values

Variables	Seven-point scales	Internal consistency*	Test-retest reliability†
Intention		0.76	0.80
- I intend to participate ...	Very unlikely/very likely		
- My plans are to participate regularly ...	Strongly disagree/strongly agree		
- I estimate that my chances to participate ...	Extremely weak/extremely good		

Attitude		0.93	0.64
I think that participating ... would be ...	Very tiresome/very stimulating		
	Very unenjoyable/very enjoyable		
	Very dull/very interesting		
	Very unpleasant/Very pleasant		
	Very bad/very good		
	Very useless/very useful		
	Very Disadvantageous/ very advantageous		
	Very harmful/very beneficial		

Subjective norm		0.83	0.59
- People who are important to me would recommend me to participate ...	Strongly disagree/strongly agree		
- People who are important to me thing I should participate ...	Strongly disagree/strongly agree		
- If you had participate ..., people who are important to you would ...	Strongly disapprove/ strongly approve		

Perceived behavioural control		0.86	0.89
- I feel capable to participate ...	Strongly disagree/strongly agree		
- I am confident that I could ...	Strongly disagree/strongly agree		
- It is completely up to me whether I participate ...	Strongly disagree/strongly agree		
- I am going to have the freedom to participate ...	Strongly disagree/strongly agree		
- For me, participating ...	Very difficult/very easy		
- How much control do you feel you have over the fact of participating ...	No control at all/complete control		

Behavioural beliefs (ten items)		0.79	0.49
- If I had participated ..., I would control better my diabetes	Very unlikely/very likely		

Normative beliefs (five items)		0.82	0.73
- My physician would disapprove-approve that I participate ...	Strongly disapprove/ strongly approve		

Control beliefs (six items)		0.86	0.78
- If I had a lack of access to facilities, I would participate ...	Very unlikely/very likely		

Anticipated regret		0.86	0.32
- If I did not participate ..., I would regret it/It would preoccupied me/It would worry me	Strongly disagree/strongly agree		

Moral Norm		0.77‡	0.43
- It is in my principles to participate ...	Strongly disagree/strongly agree		
- I would fell guilty about not to participate ...	Strongly disagree/strongly agree		

Descriptive norm			
- According to you, what proportion of individuals with diabetes participate...?	0% to 10%, 11% to 20%, 21% to 29%, etc.	-	0.66

*Note*. Internal consistency was reported as * Cronbach's alpha coefficient for variables of 3 items or more and ‡ Spearman's correlation coefficient for variables of 2 items. † = Intraclass correlation coefficient.

Past behavior was assessed as follows: "How often have you participated in one or more physical activities lasting 20 to 30 minutes per session during your free time in the last three months?" Response choices offered were 1 = never; 2 = less than once a month; 3 = two or three times a month; 4 = once a week; 5 = twice a week; 6 = three times a week; and 7 = four times a week. This method of assessing behavior is based on previous validated studies [31,32].

Age, gender, education and BMI were also assessed. Based on respondents' self-reported weight (kg) and height (m), BMI was calculated as weight (kg)/height (m).^2 ^The weight classification recommended by the WHO Expert Consultation on Obesity was used [33]; BMI between 25.0 kg/m^2 ^and 29.9 kg/m^2 ^are considered as overweight and BMI of 30.0 kg/m^2 ^or greater indicates obesity.

### Statistical Analysis

Given that there were fewer than 5% missing data related to the dependent variable intention and/or psychosocial predictors and the non-significant result in MCAR (missing completely at random) test (χ^2 ^= 296.7, *p *= 0.89), missing data were replaced by the mean substitution procedure [34]. A descriptive analysis was first performed. Second, a Pearson product-moment correlation matrix was analyzed to examine the interrelationships between the TPB determinants and additional variables. A hierarchical regression analysis was then conducted in which intention was regressed on the TPB determinants (Step 1). Thereafter, anticipated regret, moral norm and descriptive norm (Step 2), past behavior (Step 3), and age, gender, education and BMI (Step 4) were included. To remain in the final regression equation, a given variable had to reach the standard statistical significance level (*p *< .05) and, based on the squared semi-partial correlation, its additional contribution explained the variance needed to account for at least 1% of variance.

Subsequently, von Haeften's et al. [35] guidelines were applied to identify critical beliefs to be targeted for a behavior change intervention. Thus, for the TPB determinants contributing independently to the prediction of intention, the Pearson product-moment correlation matrix was analyzed: (1) to verify if belief-based measures were associated with their respective main constructs (i.e., attitude-behavioral beliefs [Σb/n], subjective norm-normative beliefs [Σn/n], perceived behavioral control-control beliefs [Σc/n]); (2) to identify key beliefs significantly correlated with intention. Then, within each belief-based measure, significant key beliefs were entered in a regression analysis to identify those making an independent contribution to the prediction of intention. As a final step, all of the significant beliefs within a belief-based measure that made an independent contribution to the prediction of intention were entered into a final regression. SPSS version 12.0 statistical software was used for all analyses.

## Results

### Descriptive Statistics and Correlation Matrix

The mean age of participants was 56.5 years (SD = 6.5), 43% were male and 26% had completed postsecondary education. Mean scores and standard deviations of psychosocial and external variables are presented in Table 2. Generally, the means of psychosocial variables were slightly positive, suggesting that participants in this study had a positive predisposition towards the regular practice of physical activity. The mean score for past behavior was 4.1 ± 2.2, indicating that, on average, participants were active about once a week during the previous three months.

**Table 2:** Correlation matrix of the variables

	INT	ATT	SN	PBC	BB	NB	CB	DN	MN	AR	PB	AGE	EDU	GEN	BMI
Mean	5.54	5.98	6.12	5.63	5.74	6.34	5.43	4.37	5.45	5.44	4.06	56.48	2.07	1.57	36.29
St. Dev.	1.42	1.07	1.05	1.26	.83	.90	1.19	1.95	1.47	1.47	2.19	6.45	.85	.49	8.24
INT	-														
ATT	.65‡	-													
SN	.45‡	.46‡	-												
PBC	.74‡	.65‡	.47‡	-											
BB	.34‡	.39‡	.43‡	.37‡	-										
NB	.45‡	.50‡	.73‡	.58‡	.51‡	-									
CB	.71‡	.65‡	.46‡	.80‡	.36‡	.51‡	-								
DN	.28‡	.23‡	.23‡	.22‡	.09‡	.17‡	.23‡	-							
MN	.69‡	.59‡	.46‡	.59‡	.32‡	.43‡	.60‡	.32‡	-						
AR	.54‡	.48‡	.40‡	.49‡	.30‡	.36‡	.57‡	.25‡	.57‡	-					
PB	.47‡	.40‡	.18‡	.48‡	.08*	.18‡	.45‡	.21‡	.40‡	.29‡	-				
AGE	.05	-.02	.00	.08*	.01	.02	.04	.06	.06	.07	.03	-			
EDU^a^	.05	.08*	.01	.04	.12**	.10*	.08*	-.06	.01	.01	-.04	-.10*	-		
GEN^b^	.01	.03	-.08*	-.01	-.01	-.09*	.00	.03	-.04	-.01	.04	-.08*	-.07	-	
BMI	-.03	-.06	.07	-.05	.14†	.10*	-.08	-.06	-.06	-.07	-.16‡	-.12†	.05	-.11**	-

*Note*. **INT **= intention. **ATT **= attitude. **SN **= subjective norm. **PBC **= perceived behavioural control. **BB **= behavioural beliefs. **NB **= normative beliefs. **CB **= control beliefs. **DN **= descriptive norm. **MR **= moral norm. **AR **= anticipated regret. **PB **= past behaviour. **GEN **= gender. **EDU **= education. **BMI **= body mass index.

a = 1 (less than high school), 2 (completed high school), 3 (completed college), 4 (completed university).

b = 1 (male), 2 (female).

* p < .05.

** p < .01.

† p < .005.

‡ p < .0001.

The matrix of correlation coefficients between intention and its determinants indicated that intention was positively correlated with all variables with the exception of age (r = .05, p = .15), gender (r = .01, p = .42), education (r = .05, p = .14), and BMI (r = - .03, p = .25). Because their *p *value was above .20, gender and BMI were not retained in subsequent analyses.

### Regression Analysis

A hierarchical regression analysis showed that attitude (β = .27, p < .0001), subjective norm (β = .09, p < .05) and perceived behavioral control (β = .52, p < .0001) accounted for 59.7% of the variance in intention (F(3,497) = 245.5, p < .0001) (Table 3). The addition of anticipated regret (β = .08, p < .05), moral norm (β = .31, p < .0001), and descriptive norm (β = .03, ns) added 7% of the variance explained in intention (ΔF(3,494) = 34.83, p < .0001); attitude (β = .15, p < .0001) and perceived behavioral control (β = .41, p < .0001) were also significant, whereas subjective norm did not reach the significance level.

**Table 3:** Summary of the hierarchical regression analysis of intention

Variable	β	R^2^	Change in R^2^	*sr*^*2*^
*Step 1*				
Attitude	.27‡			.04
Subjective norm	.08*			.004
Perceived Behavioural Control	.52‡	.597	-	.15
*Step 2*				
Attitude	. 15‡			.01
Subjective norm	.01			.000
Perceived Behavioural Control	.41‡			.08
Descriptive Norm	.03			.001
Moral norm	.31‡			.05
Anticipated regret	.08*	.667	.07	.004
*Step 3*				
Attitude	. 14‡			.01
Subjective norm	.02			.000
Perceived Behavioural Control	.38‡			.06
Descriptive Norm	.02			.000
Moral norm	.29‡			.04
Anticipated regret	.08*			.003
Past Behaviour	.09†	.673	.006	.005
*Step 4*				
Attitude	. 14‡			.01
Subjective norm	.02			.000
Perceived Behavioural Control	.38‡			.06
Descriptive Norm	.02			.000
Moral norm	.29‡			.04
Anticipated regret	.08*			.003
Past Behaviour	.09†			.005
Age	.00			.000
Education	.02	.674	.001	.000

*Note*. β = standardized regression coefficients. sr^2 ^= squared semipartial correlation.

* p < .05. † p < .005. ‡ p < .0001.

The addition of past behavior was statistically significant (ΔF(1,493) = 8.4, p < .005) but attitude (β = .14, p < .0001), perceived behavioral control (β = .38, p < .0001), anticipated regret (β = .08, p < .05) and moral norm (β = .29, p < .0001) remained additional significant determinants. However, the single contribution of anticipated regret and past behavior was lower than 1%; thus, they were not kept in the final model. None of the demographic variables made a significant contribution. In summary, attitude, perceived behavioral control and moral norm were the determinants of intention, explaining 67.3% of its variance.

### Key Beliefs Underlying Intention

Given that attitude and perceived behavioral control were found to be significant determinants of intention, their underlying beliefs were analyzed to identify critical targets for intervention. As can be seen in Table 2, correlational analyses showed that the behavioral and control beliefs were significantly correlated with attitude (r = .39, p < 0.0001) and perceived behavioral control (r = .80, p < 0.0001), respectively. In Table 4, individual correlational analyses showed that eight of the ten behavioral beliefs were significantly correlated with intention (r = .15 to .34). These significant behavioral beliefs were then entered into a regression analysis and only "makes me feel good mentally" (β = .18) and "improves my fitness level" (β = .14) contributed independently to the prediction of intention. Similarly, as can be seen in Table 4, all six control beliefs were found to have a significant relationship with intention (r = .32 to .63). Regression analysis showed that five of these beliefs contributed independently to the prediction of intention, that is "financial constraints" (β = .26), "health problems" (β = .21), "lacking time" (β = .17), "lacking access to facilities" (β = .13), and "working schedule" (β = .12).

**Table 4:** Mean and standard deviation of beliefs, and correlation coefficient with intention

Beliefs	M	SD	r
Behavioural beliefs			
Physical fitness	6.34	1.22	.33*
Weight control	618	1.43	.27*
Diabetes control	6.32	1.29	.31*
Heart health	6.36	1.24	.33*
Time management	5.69	1.65	.16*
Improve global health	6.40	1.24	.32*
Improve mental health	6.06	1.40	.34*
Lacking time to do something else	4.21	2.10	.01
Risk of hypoglycaemia	4.19	2.20	.00
Improve insulin action	5.56	1.87	.15*
Control beliefs			
Bad or poor weather	5.00	1.79	.53*
Lacking time	5.23	1.60	.61*
Financial constraints	5.36	1.68	.63*
Lacking access to facilities	5.25	1.67	.62*
Health problems	5.11	1.66	.63*
Working schedule	6.71	1.78	.32*

20 participants had missing data on one or several items

* = p < .001.

**Figure 1:**
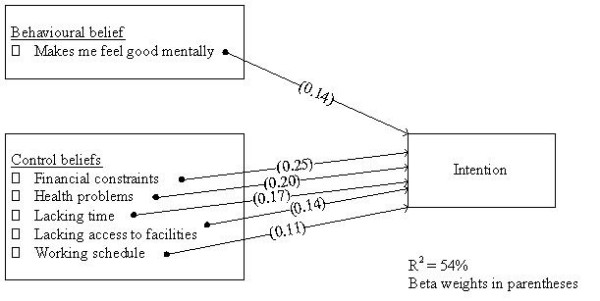
**Critical belief targets for an intervention to increase intention to participate in leisure-time physical activity of individuals with type 2 diabetes**.

Finally, to identify the critical targets for an intervention, the seven individual belief predictors identified above were entered into a final regression analysis. Figure 1 illustrates the final model containing the six critical beliefs explaining 54% of variance in intention to participate regularly in leisure-time physical activity: (1) "financial constraints" (β = .25); (2) "health problems" (β = .20); (3) "lacking time" (β = .17); (4) "makes me feel good mentally" (β = .14); (5) "lacking access to facilities" (β = .14); and, (6) "working schedule" (β = .11).

## Discussion

Using the TPB and additional determinants, the purpose of this study was to predict and explain the intention of individuals with type 2 diabetes to engage in physical activity on a regular basis. Results indicate that 67% of the variability of intention may be explained, in decreasing order of importance, by perception of control over behavior, moral norm and attitude. According to criteria established by Cohen [36], the percentage of explained variance of intention corresponds to strong effect size (*f*^2 ^≥ .35).

To our knowledge, in the context of the TPB, few studies like this one have been published on individuals with type 2 diabetes, making it difficult to place results obtained in perspective. Nonetheless, as mentioned in the introduction of this study, Plotnikoff et al. [14] found that attitude (β = 0.36, p < 0.05), subjective norm (β = 0.12, p < 0.05) and perception of behavioural control (β = 0.34, p < 0.05) explained 40% of the variance of intention to engage in physical activity among Canadians with type 2 diabetes. Other studies conducted in the field of physical activity among patients suffering from heart disease [37-39] and patients having survived cancer [40-42], revealed that 24% to 51% and 38% to 66% of the variance in intention was explained, respectively. This means that in the course of this study, results obtained explaining intention compare favorably with research carried out on symptomatic clientele. Therefore, it might be suggested that psychosocial determinants identified in this study could be used to develop an educational intervention aimed at encouraging the practice of physical activity among individuals with type 2 diabetes. The intervention would target persons exhibiting little or no motivation to adopt the practice of regular physical activity. As is now acknowledged in the scientific literature [43,44], it is advisable to distinguish a motivational phase (weak intention) from a post-motivational phase (strong intention) during the development of an educational intervention.

When considering developing an educational intervention for individuals with type 2 diabetes, particular attention should be paid to the perception of control over behavior. Indeed, this determinant has the greatest relative importance in the modeling of intention. In other terms, the decision of persons with type 2 diabetes to engage in physical activity on a regular basis is based primarily on the perceived ease of practicing such behavior. These results are congruent with observations reported for studies carried out on symptomatic populations [38,40,42,45,46]. By and large, according to Ajzen [47], the greater the relative contribution of a determinant, the greater the probability of a change to this determinant influencing intention and behavior. Thus, from a practical perspective, when the goal consists of fostering the adoption of regular physical activity among individuals with a low intention, prioritizing educational messages aimed at eliminating perceived obstacles might be suggested. Given this, five of the six obstacles identified in this study might be the target of such messages. In this respect, strategies recommended by Bandura [48], such as actively experiencing control, verbal persuasion, etc., could prove useful in achieving the above goal.

Moral norm was the second determinant of relative importance in the modeling of intention, in conformity with one of the hypotheses advanced. To date, use of the moral norm among symptomatic populations had been rather limited. In fact, to our knowledge, only one study has explored this determinant among individuals suffering from heart disease and results pointed to a non-significant contribution to the modeling of intention [37]. In the case of individuals with type 2 diabetes, the results of this study suggest that the feeling of moral obligation to engage in physical activities on a regular basis is a significant component of the decision to take action. From a practical standpoint, instilling greater motivation to regularly practice physical activities through the development of an educational message based on moral norm poses a dual challenge. First, contrary to perceived behavioral control for which the TPB offers guidelines on the definition of content of educational messages by targeting control beliefs, guidelines defining content targeting moral norm are rather limited. Indeed, in his theory of interpersonal behavior, Triandis [49] provides a rather vague idea of this construct without specifying its nature or dimensions (quoted by Blondeau et al. [50]). Likewise, Jackson et al. [22] also point to the challenge of defining the content of educational messages within acceptable ethical boundaries. For example, when promoting the regular practice of physical activity, developing a message based on «victim-blaming » would be contrary to ethical principles.

Based on the preceding, one avenue to explore to ensure the proper development of an educational message is the premise that moral norms are rooted in social norms, and when these norms become strongly anchored within a person's psyche, they have an impact on behavior irrespective of the immediate social context [20]. Concretely, since health professionals treating individuals suffering from diabetes often recommend physical activity [23], it is plausible that such recommendations might become interiorized within a certain time. Thus, the feeling of moral obligation to adopt the behavior might emerge, regardless of the immediate social context.

Although the contribution of attitude was moderate in the modeling of intention, it warrants consideration, nonetheless, in the development of an intervention. Research conducted among individuals with heart disease also reported a moderate but significant contribution of this determinant in the modeling of intention [38,39]. On the other hand, based on results observed for Canadians with type 2 diabetes [14] and different types of cancer [45,51-54], a more important contribution of attitude was reported. In this study, the moderate contribution of attitude may be explained by the fact that the participants were well aware of the advantages of regular physical activity associated with a disease such as type 2 diabetes. A review of the scores recorded on the attitude scale (scale of 1 to7) for participants with a weak (M = 4.94) or strong (M = 6.34) (data not shown) intention tends to support the explanation offered. This acknowledgement of an understanding of the beneficial effects of physical activity among individuals with type 2 diabetes has also been observed elsewhere [13,23,55].

To develop a positive attitude towards regular physical activity, educational messages might possibly be developed to briefly remind individuals of the benefits of physical activity from a psychological perspective. Indeed, among the behavioral beliefs indentified in this study that messages might explore, only psychological wellbeing proved significant in the modeling of intention. As has been demonstrated by Fishbein et al. [56], the transition from a given behavioral belief of « somewhat positive » to « very positive » may result in a significant impact on intention to adopt a given behavior.

Likewise, as reported in other studies on physical activity, the determinants of anticipated regret [17,57] and past behavior [37-39] proved significant in the modeling of intention. From a practical standpoint, however, and given their marginal contribution (< 1%) to variance explained by intention, we do not consider it relevant to develop educational messages based on either determinant. Descriptive norm, on the other hand was not significant in the modeling of intention. Several factors may explain this result. First, according to Rimel & Real [25], the greater the prevalence of a behavior within a given group, the greater the probability that said behavior will be adopted by its members in response to the prevailing norm. Yet, in this study, it was estimated that only 41% to 50% of individuals with type 2 diabetes engaged in physical activity on a regular basis. This prevalence might be insufficient to create a favorable descriptive norm. Then again, the use of one item alone to measure this construct could explain the result obtained. Finally, it is also possible that the average age (56.5 ± 6.5 years) of participants in this study might explain the non-significant contribution of descriptive norm, insofar as adults of a certain age are less likely to be swayed by this form of social pressure than younger individuals [26].

In retrospect, although the results of this study suggest avenues to explore for the development of educational messages to encourage regular physical activity among individuals with type 2 diabetes certain limitations warrant attention. First, caution should be exercised before generalization of the results to other individuals of the same age range with type 2 diabetes for two reasons: a) the response rate was low at 33%; and b) the proportion of men and women did not correspond to the reference Canadian population. In our sample more women than men participated. Nonetheless, our results are in agreement with previous studies of physical activity based on the TPB. For instance, considering only the TPB variables, Plotnikoff et al. showed among a representative sample of individuals with type 2 diabetes living in Alberta, Canada, results similar to those of the present study (see Table 3). A second limitation is that the participants in the study were possibly more interested in the subject of the study (practicing physical activities) than non-participants. A third limitation resides in the use of self-reported measurement as an indicator of practicing physical activities. However, the measurement was validated [31,32] and deemed pertinent within the context of an independent evaluation. A fourth limitation consists of the cross sectional nature of the study; intention was the outcome variable instead of prospective behavior. Although it is generally acknowledged in the field of physical activity that intention is an important determinant of future behavior[29], it remains to be determined if intention is an important and significant determinant of behavior for this population.

## Conclusion

This study identified three psychosocial determinants that explain more than two thirds of the intention to engage in physical activity on a regular basis among individuals with type 2 diabetes. A summary of these key determinants is presented in Table 5 in order to develop interventions aimed at increasing the motivation of individuals with type 2 diabetes to regularly practice physical activities.

**Table 5:** Summary of the key findings regarding factors to take into account with respect to physical activity promotion in French Canadians with type 2 diabetes

Factors	Intervention objectives	Intervention targets
Perceived behavioural control	Develop strategies to overcome regular physical activity barriers.	-Financial constraints -Health problems -Lacking time -Lacking access to facilities -Working schedule
Moral Norm	Develop a sense of moral obligation to engage in regular physical activity.	-Testimonials from health professionals to highlight the importance of regular physical activity on glycemic control
Attitude	Identify positive outcomes related to regular physical activity.	-Mental health

## Competing interests

The authors declare that they have no competing interests.

## Authors' contributions

FB and GG conceived the study design. FB carried out the study and drafted the manuscript. GG helped with data analysis and data interpretation, and provided a critical review of the manuscript. Both authors approved the final version of the manuscript.
